# Risk of Depressive Disorders Following Myasthenia Gravis: A Nationwide Population-Based Retrospective Cohort Study

**DOI:** 10.3389/fpsyt.2019.00481

**Published:** 2019-07-09

**Authors:** Hsuan-Te Chu, Chih-Chieh Tseng, Chih-Sung Liang, Ta-Chuan Yeh, Li-Yu Hu, Albert C. Yang, Shih-Jen Tsai, Cheng-Che Shen

**Affiliations:** ^1^Department of Psychiatry, Beitou Branch, Tri-Service General Hospital, National Defense Medical Center, Taipei, Taiwan; ^2^Graduate Institute of Medical Sciences, National Defense Medical Center, Taipei, Taiwan; ^3^Department of Psychiatry, Tri-Service General Hospital, National Defense Medical Center, Taipei, Taiwan; ^4^Department of Psychiatry, Taipei Veterans General Hospital, Taipei, Taiwan; ^5^School of Medicine, National Yang-Ming University, Taipei, Taiwan; ^6^Institute of Brain Science, National Yang-Ming University, Taipei, Taiwan; ^7^Division of Interdisciplinary Medicine and Biotechnology, Beth Israel Deaconess Medical Center, Harvard Medical School, Boston, MA, United States; ^8^Department of Psychiatry, Chiayi Branch, Taichung Veterans General Hospital, Chiayi, Taiwan

**Keywords:** depression, myasthenia gravis, economic condition, comorbidity, retrospective cohort study

## Abstract

The chronic autoimmune disease myasthenia gravis (MG) is characterized by fluctuating muscle weakness, which can lead to a large amount of stress in the patient. The current investigation plans to assess the risk of depressive disorders in MG patients. A retrospective cohort study of patients ageing 20 years and older and also newly diagnosed with MG between January 1, 2000, and December 31, 2008, was conducted from the National Health Insurance Research Database (NHIRD) in Taiwan. Observations of all 349 MG patients and 1,396 control individuals were made until a diagnosis of a depressive disorder by a psychiatrist, until death, or until December 31, 2013. A range of comorbidities were found, such as coronary artery disease, hypertension, diabetes mellitus, and dyslipidemia, with cerebrovascular disease being reported more frequently in MG patients in comparison with control subjects. After adjustment of patients’ sex, age, urbanization, comorbidities, and monthly income, results indicated that MG individuals are 1.94 times more at risk (95% confidence interval [CI], 1.15–3.27, *P* = 0.014) of developing depressive disorders than are controls. This showed an increased risk in the development of depressive disorders in people with MG. Thus, depressive symptoms in MG patients should be regularly assessed.

## Introduction

Myasthenia gravis (MG), one of the chronic immune diseases, is described with symptoms of unpredictable fluctuating weakness and fatigue in neck muscles, eye muscles, limb muscles, swallowing, chewing, breathing, and talking throughout the day by having an impact on the skeletal neuromuscular junction. Symptoms are usually lighter during the morning hours, worsen as the day progresses and also with exertion, but improve with rest (1).

The concurrent reduction in skeletal muscle contractions in the pathophysiology of MG is due to the antibodies directed to nicotinic acetylcholine receptors and other proteins, changing the motor circuits (2). MG has a disability prevalence of 6/100,000 (3). In a previous study using the National Health Insurance Research Database (NHIRD) to investigate the epidemiology of myasthenia gravis in Taiwan, the average annual incidence was 2.1/100,000. The prevalence increased steadily during the study period from 8.4/100,000 in 2000 to 14.0/100,000 in 2007 (4). MG is a life-long and remitting/relapsing disease with an unpredictable course (3). The cholinergic system plays a key role in memory impairment and sleep disorders as described in MG (5, 6). On the other hand, continued treatment and chronic symptoms may lead to significant restraint and diminution of health-related quality of life (7–10).

Depressive disorder has a lifetime prevalence of 7% to 17% in the general population (11, 12). In a study using NHIRD to investigate the epidemiology of depressive disorder in Taiwan, the annual incidence of depressive disorder was 3.91/1,000 (13). The fact that frequency of depression increases significantly in chronically ill individuals, a well-known significant limitation in physical functioning, was also associated with depression. Other authors have found that depression has significant impacts on the course and outcome of medical illnesses (14), including increased ambulatory visits (15), more somatic complaints (16), and reduced motivation for self-care (17, 18). It may be difficult for depression patients to get used to the aversive manifestations of chronic medical illness. With control of the severity of physical illnesses, around 50% increase of the medical costs for chronic illnesses is still associated with depression. Therefore, clinicians should pay particular attention to depression in patients with chronic medical illnesses.

Brain functions determining behavioral changes such as suicidal behaviors and depressive disorders may be affected by MG (19). In previous studies in Taiwan using NHIRD, patients with neurological disorders such as cluster headache (5.6 times) (20), Parkinson disease (4.1 times) (21), and Tourette syndrome (4.9 times) (22) are found to have a higher risk of developing depression during the follow-up period. Furthermore, psychological reactions may be predicted in patients diagnosed with MG as this disease is attenuating, chronic, and life-threatening with unpredictable progression (23). Just like patients with other chronic diseases, MG patients may possess a wide range of social and psychological disabilities that may be more critical than defective physical functions (24–26). Often, psychiatric consequences such as depressive and anxiety disorders are the result of chronic inflammatory disease conditions (3, 23, 27–29). In our previous studies using NHIRD, for example, the incidence rate of depressive disorder was higher in patients with ankylosing spondylitis (5.48 per 1,000 person-years) than in control patients (3.29 per 1,000 person-years) (28). With regard to MG, 74 patients with MG examined by Magni et al. revealed that 22% had adjustment disorder with mixed emotional features and depressed mood and that 14% matched the Diagnostic and Statistical Manual of Mental Disorders, Third Edition (DSM-III) criteria for an affective disorder (30). Considerations regarding careful psychiatric/psychological evaluation may look to the temporal association between depressive disorders and MG. However, none of the studies investigating the association between MG and the subsequent risk of depressive disorders had used large databases for such research. Therefore, we used the Taiwan National Health Insurance Research Database (NHIRD) to assess whether MG patients are at a higher risk for developing depressive disorders. We also performed a Cox proportional-hazards regression model to identify risk factors that predicted depressive disorders in the MG patients.

## Method

### Data Sources

Instituted in 1995, the NHI program is a mandatory health insurance program offering comprehensive medical care coverage, including outpatient, inpatient, emergency, and traditional Chinese medicine to all residents of Taiwan; the coverage rate is as high as 99% (31). The NHIRD contains comprehensive information regarding clinical visits, with prescription details and diagnostic codes based on the *International Classification of Diseases*, ninth revision, Clinical Modification (ICD-9-CM). Confidentiality of the NHIRD is maintained according to directives of the Bureau of the NHI, and NHIRD itself is managed by the National Health Research Institutes (NHRI). Longitudinal Health Insurance Database 2000 (LHID 2000), which is a subset of NHIRD created by NHRI, is the data source for our study. LHID 2000 contains all original claim data of 1,000,000 beneficiaries that were randomly sampled from the year 2000 Registry for Beneficiaries of the NHIRD, where registration data of everyone who was a beneficiary of the National Health Insurance program during the period of January 1, 2000, to January 1, 2001, were drawn for random sampling. There are approximately 23.75 million individuals in this registry. All the registration and claim data of these 1,000,000 individuals collected by the National Health Insurance program constitute the LHID 2000. The NHRI of Taiwan reported that there were no significant differences in gender distribution, age distribution, or average insured payroll-related amount between the patients in the LHID and those in the original NHIRD (National Health Insurance Research Database, Taiwan; http://nhird.nhri.org.tw/en/index.htm).

### Ethics Statement

This study was approved by the Institutional Review Board of the Taipei Veterans General Hospital (VGHIRB No.: 2018-07-016AC). As the NHI dataset contains only de-identified secondary data for research purposes, and a formal written waiver for the need for consent was issued by the Institutional Review Board of Taipei Veterans General Hospital, written consents were not obtained.

### Study Population

We selected patients aged 20 years and older who were newly diagnosed with MG between January 1, 2000, and December 31, 2008, to carry out a retrospective cohort study. MG was defined as ICD-9-CM code: 358.0. Patients diagnosed with depressive disorders were excluded (ICD-9-CM codes: 296.2X-296.3X, 300.4, and 311.X) before enrollment. For each MG patient included in the final cohort, four age-, sex-, and enrolment-date-matched control patients who were not diagnosed with MG or depressive disorder were randomly selected from the LHID 2000. The random assignment procedures were performed by SAS statistical software and were based on the random numbers that were generated from the uniform distribution.

Insurance premiums, calculated according to the beneficiaries’ total income, were used to estimate monthly income. Monthly income was divided into no income, low income [monthly income < 20,000 New Taiwan Dollar (NTD)], median income (20,000 NTD ≤ monthly income < 40,000 NTD), and high income (monthly income ≥ 40,000 NTD). Urbanization was categorized into three groups: urban, suburban, and rural. Urbanization and monthly income levels were used to represent socioeconomic status. Furthermore, the preexisting comorbidities at the date of enrolment, including hypertension, diabetes mellitus, dyslipidemia, coronary artery disease, congestive heart failure, hyperthyroidism, hypothyroidism, and cerebrovascular disease, were identified in both cohorts.

All MG and control patients were observed until a diagnosis of a depressive disorder by a psychiatrist, until death, or until December 31, 2013. The primary clinical outcome assessed was psychiatrist-diagnosed depressive disorder.

### Statistical Analyses

Demographic differences between MG and control patients were examined using independent *t*-tests and chi-squared tests. Calculation of the incidence rate (per 1,000 person-years) of newly diagnosed depressive disorders in MG and control individuals was made. To investigate potential surveillance bias, subgroups were stratified according to the duration since MG diagnosis.

Variables that predicted depressive disorder in MG and control patients as well as in MG patients only were identified using Cox proportional-hazards regression model. Covariates in the univariate model included control variables such as age; sex; common comorbidities, including hypertension, diabetes mellitus, dyslipidemia, coronary artery disease, congestive heart failure, hyperthyroidism, hypothyroidism, and cerebrovascular disease; urbanization; and monthly income. Factors that demonstrated a moderately significant statistical relationship in the univariate analysis (*P* < 0.1) were entered by forward selection in a multivariate Cox proportional-hazards regression model (32).

Data were extracted and computed using the Perl programming language (version 5.12.2). The Microsoft SQL Server 2005 (Microsoft Corp., Redmond, WA, USA) was used for data linkage, processing, and control sampling. IBM SPSS (version 19.0 for Windows; IBM Corp., New York, NY, USA) and SAS statistical software (version 9.2; SAS Institute Inc., Cary, NC, USA) were used to perform all statistical analyses. Comparisons resulting in a *P* value of less than 0.05 were considered to indicate a statistically significant relationship.

## Results

### Participant Selection

A total of 349 MG patients and 1,396 control individuals without depression were selected; among them, 53.6% were women. The median age at enrollment was 44 years [interquartile range (IQR), 32–56 years], and the median follow-up periods for the MG and control patients were 8.54 (IQR, 6.13–10.71 years) and 8.78 years (IQR, 6.46–10.99 years), respectively. Comorbidities including hypertension, diabetes mellitus, dyslipidemia, coronary artery disease, hyperthyroidism, and cerebrovascular disease were reported more frequently in the MG patients than in the control patients. **Table 1** shows the demographic and clinical variables of the MG and control patients.

**Table 1:** Baseline characteristics of patients with and without myasthenia gravis.

Demographic data	Patients with myasthenia gravis *n* = 349		Patients without myasthenia gravis *n* = 1,396		*P* value
	*n*	%	*N*	%	
Age (years)^a^	44 (32–56)		44 (32–56)		>0.999
≥65	48	13.8	192	13.8	
<65	301	86.2	1,204	86.2	
Sex					>0.999
Male	162	46.4	648	46.4	
Female	187	53.6	748	53.6	
Comorbidities					
Hypertension	95	27.2	285	20.4	0.007*
Diabetes mellitus	71	20.3	169	12.1	<0.001*
Dyslipidemia	91	26.1	221	15.8	<0.001*
Coronary artery disease	67	19.2	149	10.7	<0.001*
Congestive heart failure	10	2.9	22	1.6	0.118
Hyperthyroidism	36	10.3	36	2.6	<0.001*
Hypothyroidism	7	2.0	10	0.7	0.059
Cerebrovascular disease	58	16.6	98	7.0	<0.001*
Degree of urbanization					0.037*
Urban	250	71.6	907	65.0	
Suburban	85	24.4	397	28.4	
Rural	14	4.0	92	6.6	
Income group					0.012*
No income	73	20.9	286	20.5	
Low income	139	39.8	672	48.1	
Medium income	70	20.1	249	17.8	
High income	67	19.2	189	13.5	
Follow-up years^a^	8.54 (6.13–10.71)		8.78 (6.46–10.99)		0.053

^a^Indicates median (interquartile range). *Indicates statistical significance.

### Incidence Rate of Depression in MG and Control Cohort

During the follow-up period, 22 MG patients (7.60 per 1,000 person-years) and 43 control patients (3.55 per 1,000 person-years) were diagnosed with depressive disorders (**Table 2**). The incidence risk ratio (IRR) of depressive disorder between the MG and control patients was 2.14 (95% CI, 1.22–3.65). When stratified with the follow-up durations, higher IRR of newly diagnosed depressive disorder remained significantly increased in longer follow-up duration (≥1 year).

**Table 2:** Number of newly diagnosed depressive disorders between myasthenia gravis and control subjects, which was stratified by follow-up duration.

Follow-up duration (year)	Myasthenia gravis		Control cohort		Incidence rate ratio (95% CI)
	No. of depressive disorder	Per 1,000 person-years	No. of depressive disorder	Per 1,000 person-years	
Overall	22	7.60	43	3.55	2.14 (1.22–3.65)
0–1	4	1,017.81	4	524.24	1.94 (0.36–10.42)
≥1	18	6.22	39	3.23	1.93 (1.04–3.45)

### MG on Risks of Clinical Depression

**Figure 1:**
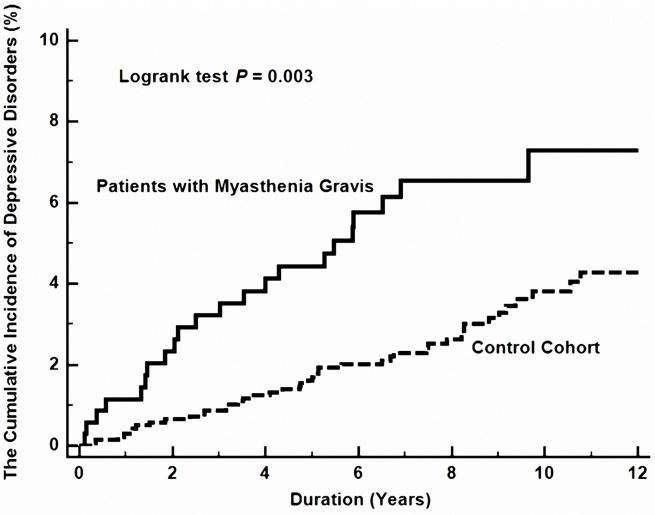
The cumulative risk of depressive disorder between patients with and without myasthenia gravis.

In MG patients, in comparison with control individuals, the hazard ratio (HR) for developing depressive disorders during the follow-up period was 1.94 times (95% CI, 1.15–3.27, *P* = 0.014) more likely, after adjustment for age, sex, comorbidities, urbanization, and monthly income (**Table 3** and **Figure 1**).

**Table 3:** Analyses of risk factors for depressive disorder in patients with and without myasthenia gravis.

Predictive variables	Univariate analysis		Multivariate analysis	
	HR (95% CI)	*P* value	HR (95% CI)	*P* value
Myasthenia gravis	2.13 (1.27–3.56)	0.004	1.94 (1.15–3.27)	0.014*
Age (<65 = 0, ≥65 = 1)	1.57 (0.82–3.01)	0.172		
Sex (female = 1, male = 0)	0.99 (0.61–1.62)	0.969		
Comorbidities				
Hypertension	1.21 (0.68–2.15)	0.523		
Diabetes mellitus	0.99 (0.47–2.08)	0.980		
Dyslipidemia	1.41 (0.78–2.55)	0.255		
Coronary artery disease	1.46 (0.74–2.86)	0.276		
Congestive heart failure	0.05 (0.00–791.17)	0.541		
Hyperthyroidism	1.62 (0.59–4.44)	0.353		
Hypothyroidism	0.05 (0.00–3,917.43)	0.601		
Cerebrovascular disease	0.68 (0.48–0.95)	0.024	0.70 (0.50–0.99)	0.044*
Degree of urbanization				
Urban	Reference			
Suburban	0.37 (0.17–0.77)	0.008	0.37 (0.17–0.78)	0.009*
Rural	1.12 (0.45–2.80)	0.813	1.25 (0.50–3.14)	0.638
Income group				
No income	Reference			
Low income	1.08 (0.57–2.07)	0.807		
Medium income	0.82 (0.36–1.86)	0.627		
High income	1.03 (0.45–2.34)	0.951		

HR indicates hazard ratio; CI indicates confidence interval. *Indicates statistical significance.

### Risks Factors for Depression in MG Patients

Variables that predicted depressive disorders in MG patients were identified by performing a Cox proportional-hazards regression model. Results showed that only medium monthly income is a favorable prognostic factor for depressive disorders in the MG patients (HR: 0.12; 95% CI, 0.02–0.94, *P* = 0.044) (**Table 4**).

**Table 4:** Analyses of risk factors for depressive disorder in patients with myasthenia gravis.

Predictive variables	Univariate analysis		Multivariate analysis	
	HR (95% CI)	*P* value	HR (95% CI)	*P* value
Age (<65 = 0, ≥65 = 1)	2.19 (0.81–5.95)	0.123		
Sex (female = 1, male = 0)	1.21 (0.53–2.80)	0.650		
Comorbidities				
Hypertension	1.36 (0.56–3.35)	0.499		
Diabetes mellitus	0.42 (0.10–1.81)	0.246		
Dyslipidemia	1.13 (0.44–2.89)	0.801		
Coronary artery disease	1.31 (0.49–3.56)	0.591		
Congestive heart failure	0.05 (0.00–19,956.41)	0.646		
Hyperthyroidism	0.87 (0.20–3.70)	0.846		
Hypothyroidism	0.05 (0.00–17,552.01)	0.642		
Cerebrovascular disease	0.69 (0.43–1.10)	0.119		
Degree of urbanization				
Urban	Reference			
Suburban	0.48 (0.14–1.64)	0.244		
Rural	1.19 (0.16–8.95)	0.864		
Income group				
No income	Reference			
Low income	0.59 (0.23–1.54)	0.282	0.59 (0.23–1.54)	0.282
Medium income	0.12 (0.02–0.94)	0.044	0.12 (0.02–0.94)	0.044*
High income	0.50 (0.15–1.65)	0.254	0.50 (0.15–1.65)	0.254

HR indicates hazard ratio; CI indicates confidence interval. *Indicates statistical significance.

## Discussion

We analyzed the risk of depressive disorder in MG patients by conducting a nationwide study. Results revealed that in MG patients, in comparison with control individuals, the HR for developing depressive disorders during the follow-up period increased to 1.94 times, after adjustment for age, sex, comorbidities, urbanization, and monthly income. It was also noted that MG patients had higher prevalence of hypertension, diabetes mellitus, dyslipidemia, coronary artery disease, hyperthyroidism, and cerebrovascular disease (**Table 1**), which are possibly related to the long-term prednisolone use, a first-choice immunosuppressive treatment, of MG patients (33, 34).

Our results revealed that MG might be a risk factor for consequent depressive disorders. Previously, the 74 patients with MG examined by Magni et al. had been found 22% to have adjustment disorder with mixed emotional features and depressed mood (30). In a previous study, depressive symptoms in MG patients were associated with disease severity, dose of oral glucocorticoids, longer duration of illnesses, and muscle weaknesses (35).

The increased risk of depressive disorders in MG patients may be due to various factors. One of the reasons may be that these patients are experiencing chronic and disabling situations such as MG, which result in restrictions in all perspectives of life. A cross-sectional study showed that the more severe the MG in individuals, the higher the likelihood of having depressive symptoms and anxiety symptoms, in comparison with that in individuals with less severe MG (36). Furthermore, treatment options for the disease may also cause psychiatric morbidity. Corticosteroids, which are common immunosuppressive agent, are currently the approved therapy for MG that is intractable to anti-cholinesterase medications (37). Depression and anxiety are associated with corticosteroid use, but corticosteroid use especially tends to induce depressive symptoms in long-term therapy (38, 39). The incidence of adverse effect is directly related to dosage (40). Therefore, dosage regulation, joint therapies, and psychiatric evaluations may be essential in coping with the unfavorable psychiatric effects of corticosteroid use. Also, it has been reported that the cholinergic effects of anticholinesterase agents play a role in the pathophysiology of depression in patients with or without MG (16). In terms of the psychological and social aspects of the individuals, patients with multi-drug therapies or more severe diseases and incapacities may have more emotional disturbances. A previous study demonstrated that stressful life events were associated with anxiety and depression, which developed in patients with MG (36).

In this study, we found that “medium income” might be a favorable prognostic factor for depressive disorders in the MG patients (**Table 3**). The marginal significance (*P* value of 0.044) suggests that the findings might be accidental. Furthermore, **Table 1** shows that the MG patients were, on average, significantly richer than the controls, which may also affect the findings. Further study is needed to exclude a spurious association.

The increased risk of depressive disorders in MG patients may be due to surveillance bias. Patients with MG are more likely to visit hospitals, thus leading to an early diagnosis of depressive disorders. To investigate potential surveillance bias, we conducted a subgroup analysis that was stratified according to the duration between the diagnosis of MG and new-onset depressive disorders (**Table 2**). When patients diagnosed with depressive disorders within 1 year of MG diagnosis were excluded, the incidence risk ratio for the newly diagnosed depressive disorder remained high for the MG cohort, and the ratio was statistically significant. Thus, this result suggests that the increased risk of depressive disorder in MG patients was not caused by surveillance bias.

There is limited published information on the psychological and pharmacological treatments for the psychiatric morbidities associated with MG. The results of the accessible studies may not be utilized as common treatment principles due to methodological limitations or small study population. Supportive and cognitive psychotherapies may be helpful in patients who had neurological disease with subsequent psychiatric symptoms that developed (41, 42). The use of psychotropic medications in MG patients had limited published researches without randomized and placebo-controlled designs. In an open clinical study, fluoxetine had weight-reducing effect found in 13 overweight patients with MG who had long-term corticosteroids treatment, although the study group had no depressed patients (43). In another 12-week open-label, prospective study trial for MG patients with major depression, the use of citalopram showed significant improvement in depression without deterioration in the progression of MG (44). Electroconvulsive therapy is an option for treating resistant affective disorders, including depressive and catatonic episodes. Electroconvulsive therapy is a workable therapeutic option in patients with MG when psychiatric problems secondary to MG or the psychotropic medications show no response (45).

Large sampling size and MG and depressive disorder diagnoses made by specialists were the strengths of our study. In addition, our study design included an unbiased participant selection process. Due to the fact that participation in the NHI was mandatory and all residents of Taiwan could reach health care with low copayments, referral biases were low and follow-up compliance was high.

Certain limitations to our findings should be considered.

1. Due to differences between individual physicians, diagnoses input into the NHI database may be diverse. In order to eliminate this limitation, MG diagnosed by neurologists and depressive disorders diagnosed by psychiatrists where each had at least two consensus diagnoses were included in the current study.
2. Some risk factors for depressive disorders were unable to be extracted from the NHI database (such as drinking habits or smoking, level of education, religious beliefs, exercise habits, and body mass index) and thus could not be included into the evaluation, which may result in a bias.
3. Underlying mechanisms of MG and depressive disorders could not be directly examined and analyzed using population-based retrospective cohort studies.
4. The severity, the subgroup of MG, and the treatment strategy for MG were unknown in our study; and whether these factors influence the risk of developing depressive disorder warrants further study.

The results of our nationwide population-based retrospective cohort study indicated that MG patients possessed a higher risk of developing depressive disorders, with their level of monthly income as a key major factor. This meant that clinicians should pay more attention to MG patients’ psychological evaluation and give suitable psychological care.

## Data Availability

The datasets for this study will not be made publicly available because the data that support the findings of this study are available from Taiwan National Health Insurance Research Database (NHIRD). To gain access, interested individuals should contact NHIRD.

## Ethics Statement

This study was approved by the Institutional Review Board of the Taipei Veterans General Hospital (VGHIRB No.: 2018- 07-016AC). As the NHI dataset contains only de-identified secondary data for research purposes, and a formal written waiver for the need for consent was issued by the Institutional Review Board of Taipei Veterans General Hospital, written consents were not obtained.

## Author Contributions

Study conception and design: H-TC, C-CT, and S-JT. Acquisition of data: AY, C-CS, and SJ-T. Analysis and interpretation of data: CC-T, CS-L, TC-Y, L-YH, and C-CS. Drafting of manuscript: H-TC, C-CS, and S-JT. All authors read and approved the final manuscript.

## Funding

This work was supported by grant V108C-038 from the Taipei Veterans General Hospital. The funders had no role in the design and conduct of the study; collection, management, analysis, and interpretation of the data; preparation, review, or approval of the manuscript; and decision to submit the manuscript for publication.

## Conflict of Interest Statement

The authors declare that the research was conducted in the absence of any commercial or financial relationships that could be construed as a potential conflict of interest.
